# On-body non-invasive glucose monitoring sensor based on high figure of merit (FoM) surface plasmonic microwave resonator

**DOI:** 10.1038/s41598-023-44435-6

**Published:** 2023-10-16

**Authors:** Farzad Soltanian, Mehdi Nosrati, Saleh Mobayen, Chuan-Chun Li, Telung Pan, Ming-Ta Ke, Paweł Skruch

**Affiliations:** 1 Department of Electrical Engineering, University of Alberta, Edmonton, Canada; 2 Department of Electrical Engineering, Manhattan College, New York, USA; 3 Department of Electrical Engineering, University of Zanjan, Zanjan, Iran; 4 Graduate School of Intelligent Data Science, National Yunlin University of Science and Technology, Douliou, 640301 Yunlin Taiwan; 5 National Taiwan University Hospital Yunlin Branch, Yunlin, Taiwan; 6 Bachelor Program in Interdisciplinary Studies, College of Future, National Yunlin University of Science and Technology, Yunlin, 64002 Taiwan; 7 Department of Automatic Control and Robotics, AGH University of Science and Technology, 30-059 Kraków, Poland

**Keywords:** Diseases, Health care, Health occupations, Medical research, Engineering

## Abstract

High-figure of merit (FoM) plasmonic microwave resonator is researched as a non-invasive on-body sensor to monitor the human body's blood glucose variation rate in adults for biomedical applications, e.g., diabetic patients. The resonance frequencies of the proposed sensor are measured to be around ${f}_{01}=3.25$ GHz and ${f}_{01}=4.67$ GHz over the frequency band of DC to 6GHz which are suitable for monitoring interstitial fluid (ISF) changing rate. The $40\times 40 {\mathrm{mm}}^{2}$ sensor is experimentally wrapped on the human body arm to monitor the blood glucose changing rate via amplitude and frequency variations of the sensor. Amplitude variation and frequency shift are measured to be around 7 dB and 30 MHz, respectively. The measured results demonstrate the high precision of the proposed approach to depict a valid diagram for glucose changing rate due to good impedance matching of the designed microwave sensor and human body. The sensor is shown to enhance the sensitivity by a factor of 5 compared to the conventional ones.

## Introduction

Diabetes is regarded as a chronic disease that affects 422 million people worldwide with swiftly increasing propagation rates^1,2^. It is associated with remarkable morbidity as one of the top ten distinguished reasons for death worldwide. Indifference to diagnosis and treatment of diabetes developing in a person can cause macrovascular and microvascular problems, e.g., stroke and diabetic nephropathy. Regular management of blood glucose to manage diabetes is vital and enables patients to discern the relationship between levels of blood glucose with meals, activities, and crucial diabetic drugs. In prevalent blood glucose monitoring methods blood samples are extracted from the fingers which can be intrusive, sore, and unsightly. In^3^, a well-developed electronic device was designed to count carbohydrates, measure blood glucose, calculate insulin dose, and show the required insulin to self-injections. Their work still suffers from being tensive due to pricking the finger to extract a droplet of blood. Nowadays, developing precise pain-free user-friendly non-invasive glucose monitoring devices is highly in demand to improve therapy and facilitate the lives of millions of diabetic people^4–6^.

The review of the literature reveals that non-invasive glucose monitoring technique can be classified into four categories including optical, electrochemical, electromagnetic (EM), and electromechanical techniques. The optical technique works based on the optical wavelength of the spectrum, such as spectroscopy^7–17^, optical change tomography^18^, temperature-modulated localized reflection^19^, polarization change^20^, fluorescence method^21^, photoplethysmography^22^, smart hologram^23^, and optical bridge^24^. The optical technique demonstrates many advantages such as light transmission, absorption, and tracing its quality through the human body, at the expense of low resolution of the signal. Moreover, electrochemical technique can be classified into different categories including body fluid analysis^25–27^, breath chemical analysis^28^, and reverse iontophoresis^29^. In body fluid analysis, the objective is to find a correlation between the quantity of blood glucose with glucose contained in sweat^25^, saliva^26^, urine^26^, and tears^27^. In the aforementioned studies, the obtained results confirm that the glucose in sweat is much more matched to the blood glucose rather than the glucose in saliva and tear, showing that the approaches suffer from the body’s ever-changing. Furthermore, the ultrasonic technique electromechanically works based on deep penetration into skin and tissue^30^. This approach demonstrates precise results by counting the blood glucose content. Nevertheless, the approach suffers temperature changes of the experiment set-up severely influence the precise results.

The initial presentation of non-invasive human glucose monitoring is reported to be based on the concept of bioimpedance spectrograph^31^. This approach works based on employing electrical current to the human body from one side and receiving it from the other side and finding related impedance according to ohm criteria. Investigations reveal that the tissues and blood containing liquid and convey ions, i.e., ${\mathrm{Na}}^{+}$, ${\mathrm{K}}^{-}$, and ${\mathrm{Cl}}^{-}$; have a distinguished role in passing current in the human body. In this approach, the glucose content is correlated with a specific resonance frequency which is proportional to the human body’s impedance. The initial results are reported to be inspiring; however, the technique enclosed a few obstacles that quickly disturb the glucose-related signal, not allowing a satisfactory tracking of glucose swings. Consequently, the technique requires auxiliary sensors to solve the problems, e.g., temperature, sweat, or perfusion changes which promote improvement toward real-time monitoring of glucose oscillations in vivo. In an empirical test of the human case, one study positioned the projected circular spiral sensor at the test subjects’ thumb and wrist to investigate the correlation between the forwarding transfer function of the sensor and increasing blood glucose levels^4^. The calibration data: i.e., frequencies between 100 MHz and 5 GHz, for predictions of the blood glucose of human volunteers, were originated by employing a combination of main component analysis and numerous regressions. In^5,6,31^, EM-based split ring-based resonators were employed for non-invasive glucose monitoring where the resonance frequency and bandwidth of a microwave resonant circuit depend on the dielectric properties of the tissues. Although the technique is a novel work, no data is reported regard to the measurement resolution. In^32–34^, a Whispering Gallery Mode sensor was exposed on the arm skin tissue to monitor changes in blood glucose level. The reported measurement resolution in^32–34^ was 10 mg/dL of real blood glucose level. In^35^, a multi-layered EM-based structure was proposed for the human body that contains three layers of skin, fat, and muscle. In^36^, a heterogeneous structure was proposed to model the human tissues in the near sensor/antenna to consider near filed sensor behavior.

In this study, we found that the human body model described in^36^ can be suitable to investigate the blood glucose change. Electromagnetic waves are used to monitor the glucose in the fluid between the human skin cells or interstitial fluid. Surface plasmonic resonator sensor is designed to resonate in the limited frequency band; i.e. 3.25 GHz and 4.67 GHz, which is suitable for monitoring interstitial fluid (ISF) rate change due to the fact that the human body can absorb considerable EM energy in the frequency range of 300 MHz to several GHz^37,38^. To do this, a three-step experiment is designed to check the accuracy of the designed sensor glucose monitoring. In the experiment, we ask the volunteer to eat 200 g of honey after his ponderous sport to measure the drastically changing rate of glucose in the human body. According to our knowledge, the allowed daily honey consumption for a healthy adult man is 50 g. In addition, consuming 4 g of honey per kilogram of body weight is allowed for an athlete.

This paper is organized as follows: in “Microwave plasmonic Fano resonance sensor”, a microwave sensor is designed and analyzed for glucose monitoring. In “Senor design and characterization”, a model of the body is initially proposed, showing that the designed resonator can be used for blood glucose monitoring by finding the suitable resonance frequency. Moreover, an experiment is set up to test the efficiency of the proposed sensor, evaluating the performance of the sensor for different cases. Finally, the paper’s conclusion is presented in “Conclusions and future works”.

## Microwave plasmonic Fano resonance sensor

**Figure 1:**
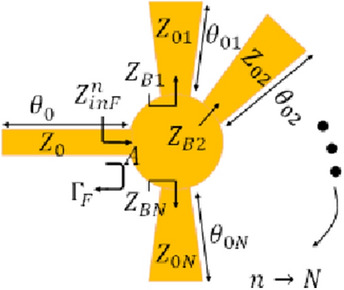
Schematic of plasmonic Fano-resonance sensor with *N* stubs.

Microwave resonators not only are used for communication systems^39–44^, but they are also used as sensors for multiple applications^45–48^. Compared to the conventional microwave resonators, Surface plasmonic resonators are reported as highly sensitive resonators by which the EM waves can strongly interact with the surrounding medium^49–51^. Here, this type of microwave resonator is considered for glucose monitoring. Figure 1 shows a diagram of a plasmonic Fano-resonance sensor with N stubs. The sensitivity of the sensor is initially analyzed using the reflective phase of the structure, compared to the conventional ones and the performance of the sensor is experimentally researched.

Similar to the analytical procedure in^52^, the sensitivity of a plasmonic Fano resonance (PFR) sensor is computed. To compute the sensitivity, the reflection coefficient ${\Gamma }_{F}$ is calculted at point A between the input feeding line and the PFR. The input impedance of PFR can be calculated in Eq. (1)

1 $${Z}_{inF}^{n}=-j({\overline{Z} }_{B}/n)\mathrm{cot}({\Theta }_{B})$$

where ${\overline{Z} }_{B}$ stands for the characteristic impedance of each stub in the PFR ${\overline{Z} }_{B}={\overline{Z} }_{B1}={\overline{Z} }_{B2}={\overline{Z} }_{Bn}$, $n\to N$, and normalized to the characteristic impedance of the feedline Z_0_. Moreover, ${\Theta }_{B}={\theta }_{01}={\theta }_{02}={\theta }_{0n}$, $n\to N$*.*

Similarly, the reflection coefficient of the PFR is calculated as follows:

2 $${\Gamma }_{F}=\frac{1+j({\overline{Z} }_{B}/n)\mathrm{cot}({\Theta }_{B})}{-1+j(\frac{{\overline{Z} }_{B}}{n})\mathrm{cot}({\Theta }_{B})}$$

where the feedline with reference characteristic impedance Z_0_ is not considered^52^. The phase of the reflection coefficient is then computed in Eq. (3).

3 $${\theta }_{{\Gamma }_{F}}=2arctan\left((\frac{{\overline{Z} }_{B}}{n})\mathrm{cot}({\Theta }_{B})\right)$$

Finally, the sensitivity of the PFR sensor to the phase of the sensitive region (patch) or ${\theta }_{s}$, defined as ${S}_{{\theta }_{s}}^{CF}$, can be computed in Eq. (4).

4 $${S}_{{\theta }_{s}}^{CF}=\frac{\partial {\theta }_{{\Gamma }_{CF}}}{\partial {\theta }_{s}}=-\frac{{(\overline{Z} }_{B}/n){CSC}^{2}({\theta }_{s})}{{\left({\overline{Z} }_{B}/n\right)}^{2}{cot}^{2}(({\theta }_{s}))+1}$$

**Figure 2:**
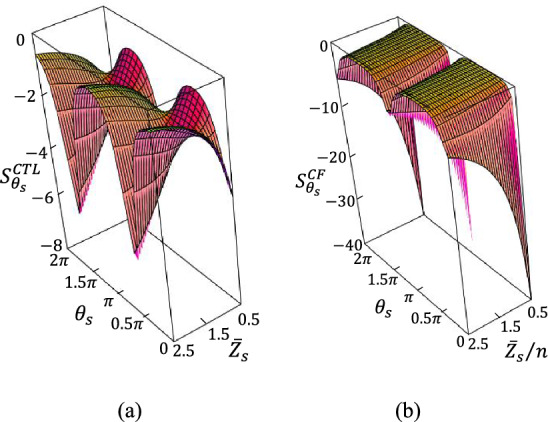
3D plots of sensitivity of the (**a**) conventional stepped impedance resonator (SIR) (function of normalized characteristic impedance and electrical length of the sensitive region)^52^ and (**b**) PFR sensors.

Figure 2 compares the 3D plot of the sensitivity of the conventional SIR in^52^ and the PFR sensors in terms of the normalized characteristic impedance and electrical length or phase of the sensitive region.

The theoretical results in Fig. 2 show the maximum sensitivity can be enhanced up to a ratio of 5 by the PFR sensor.

## Senor design and characterization

**Figure 3:**
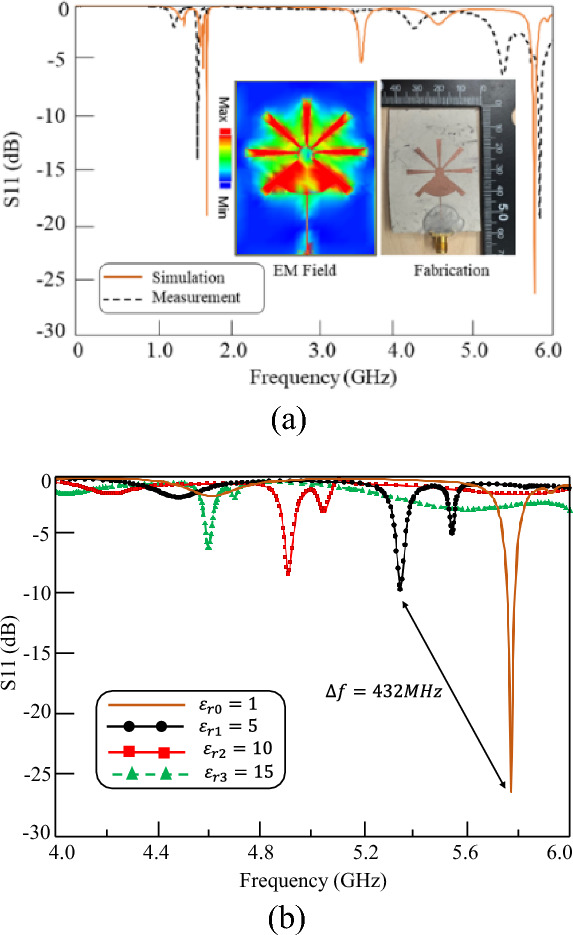
(**a**) Simulated and measured performances of ( ${\mathrm{S}}_{11})$ and a digital photograph and simulated current distribution of the PFR. (**b**) EM performance of the sensor for different materials, ${\varepsilon }_{r0}=1 \left({f}_{r0}=5.770GHz\right)$, ${\varepsilon }_{r1}=5 \left({f}_{r1}=5.338GHz\right)$, ${\varepsilon }_{r2}=10 \left({f}_{r2}=4.909GHz\right)$, and ${\varepsilon }_{r3}=15 \left({f}_{r3}=4.595GHz\right).$

The PFR is designed and fabricated, and its performance is measured without any MUT. Figure 3a shows the simulated and measured performance accompanied by a digital photo of the fabricated of the PFR. The PFR is fabricated on a 50-mil Rogers 6010 substrate with a dielectric constant of 10.2. The performance of the structure is also simulated for different MUTs. Figure 3b shows the EM results of the PFR structure as a sensor (PFRS) for MUT with different dielectric constant. The PFRS is simulated for different dielectric constants of air (${\varepsilon }_{r0}=1$), ${\varepsilon }_{r1}=5$, ${\varepsilon }_{r2}=10$, and ${\varepsilon }_{r3}=15$ (the thickness of MUTs is chosen to be 1.27 mm with loss tangent of 0.0027)where the resonance frequency of the structure is shifted toward the lower frequencies as provided in the caption of Fig. 3. There are some important characteristics for a sensor including sensitivity, frequency detection resolution (FDR), and quality factor^53–55^ where Table 1 compares the performance of PFRS with that of the conventional single-port ones. Inspecting the results, the proposed PFRS enhances the performance of such sensors.

**Table 1:** Comparison of the proposed sensor with conventional sensors.

	${f}_{0}$ $\left(\mathrm{GHz}\right)$	Sensitivity (%)	FDR ($\mathrm{GHz}$)	Q
^56^	7.720	0.91	0.06	436
^57^	9.160	1.34	0.805	753
This work	5.770	1.87	0.108	962

### A On-body test results

In this section, the designed resonator in “Microwave plasmonic Fano resonance sensor” is shown to be suitable for blood glucose monitoring. An experiment is designed for blood glucose monitoring. Based on an EM simulation tool (HFSS-Ansys), the thickness of the heterogeneous multi-layered model of body layers consisting of skin, fat, and muscle are taken as 4, 20, and 10 mm, respectively^58^. Table 2 provides the electrical properties of the three layers in human body phantom tissues^58^.

**Table 2:** Electrical properties of human body phantom tissues^58^.

Biological tissue	Permittivity ($\varepsilon^{\prime}$*r*)	Conductivity (σ, S/m)
Fat tissue	5.560525	0.041934
Skin	45.753101	0.708836
Muscle	58.482101	0.851437

**Figure 4:**
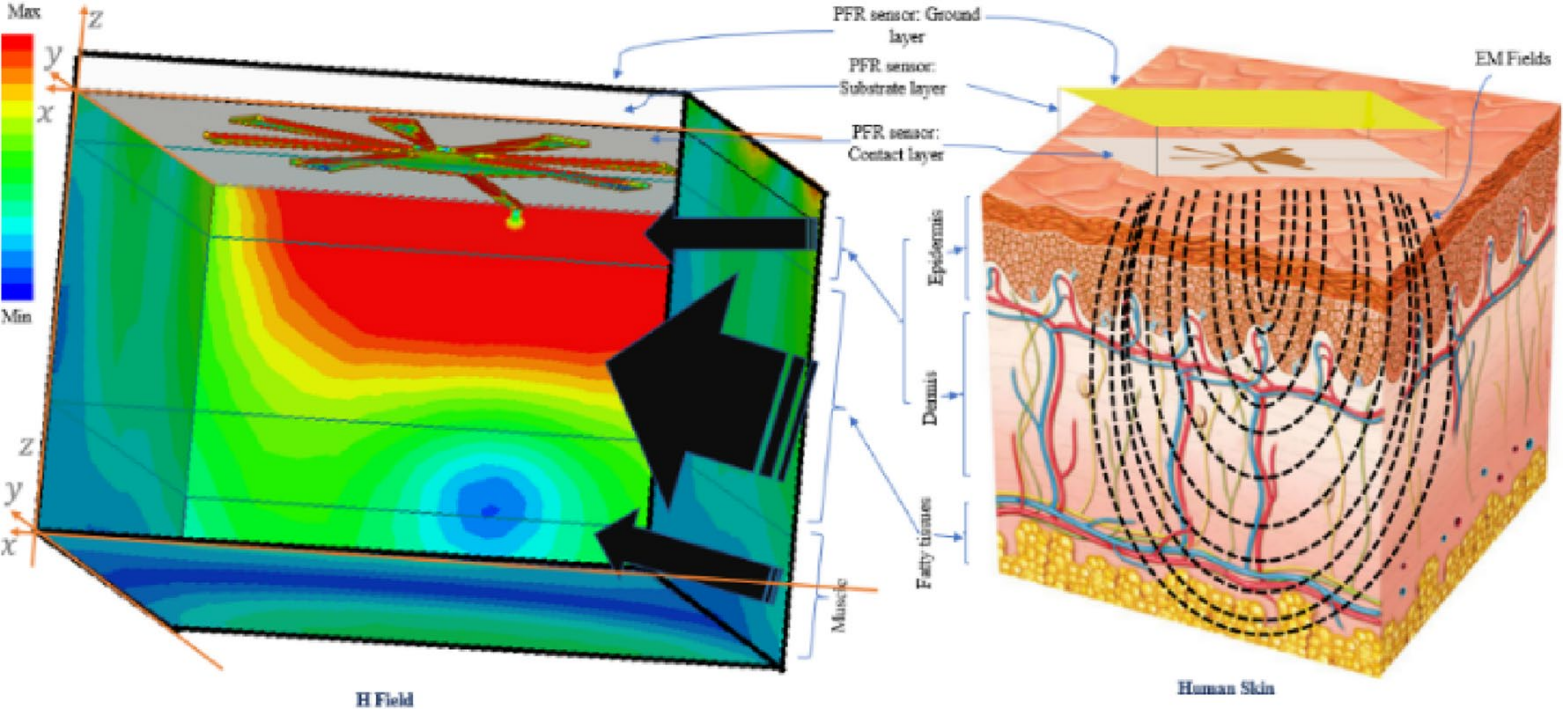
A representation of the simulated PFR sensor in contact with human skin (anatomic representation of human skin^59^) and the electromagnetic field concentration on the three layers of the human body (skin, fatty tissues, and muscle). Simulated results show that the human skin layer has more influenced by the EM fields of the PFR sensor rather than other layers.

The human skin is composed of three main layers, i.e., epidermis, dermis, and subcutaneous tissue^59^. The epidermis approximately has 100µm thick and the highly vascularized layer, i.e., the dermis, has a thickness of 1 up to 3 mm^59^. An anatomic model of human body skin is represented in Fig. 4 to analyze the changes in the frequency and the amplitude of the EM fields on the PFR sensor penetrating the different layers of human skin. The epidermis and the dermis layers contain the most percentage of the whole interstitial fluid (ISF) where the simulated results show that the EM fields are mostly concentrated in this area. As shown in Fig. 4, PFR sensor is used for monitoring the blood glucose changes in this area.

### B Experiment details

**Figure 5:**
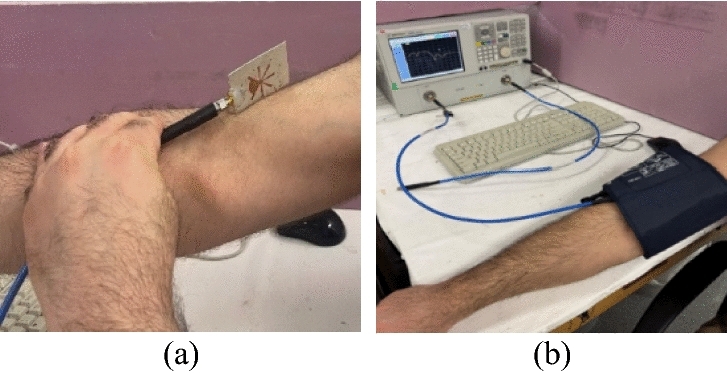
(**a**) PFR sensor placement on the patient’s arm. (**b**) Complete measurement PFR sensor setup .

In this subsection, an experiment is carried out for monitoring the blood glucose changes in the human body case. This experiment is designed based on the PFR sensor which is fixed on the volunteer’s right arm. As shown in Fig. 5a, the PFR sensor is placed on the right arm in such a way that the resonator attaches the patient’s arm, and the ground of the structure is upward. Next, the PFR sensor is wrapped by a blood pressure cuff as shown in Fig. 5b. The designed experiment consists of three steps as follows. First, resonance frequencies related to the human body's blood glucose rate are recorded with no body exercise nor any movement of the volunteer person. Next, the shifts in the resonance frequencies are monitored and recorded during the volunteer’s warming up the left-hand with, dumbbell for 10 min. Then, in the last part of the experiment, the volunteer gradually eats 200 g of honey which takes about 10 min. It should be noted that since the volunteer for the designed experiment was an adult man who did not have any diabetic disease and his weight is 89 kg, the maximum permitted daily honey consumption for him easily can be calculated by multiplying 89 by 4 g.

### C The experiment results and discussion

**Figure 6:**
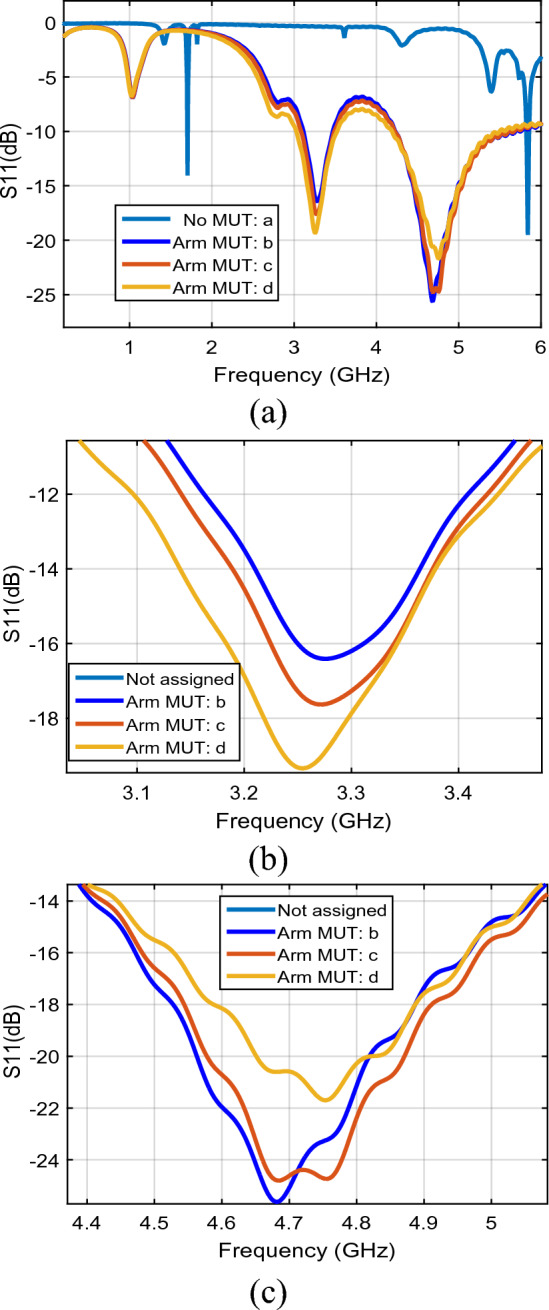
The return loss (${\mathrm{S}}_{11}$) per frequency for (**a**): first (${f}_{01}$) and second (${f}_{02}$) resonance frequencies, (**b**) first resonance frequency (${f}_{01}$), (**c**) second resonance frequency (${f}_{02}$) (line a: no MUT, line b: arm as MUT with no activity, line c: arm as MUT after 1 min activity, line d: arm as MUT 3 min after starting eating honey).

The blood glucose is monitored by tracking the changes in the return loss of electromagnetic waves ($${S}_{11})$$, whose changes are proportional to the human blood glucose changes. Figure 6a shows the $${S}_{11}$$ in the different sampling conditions consisting of Air as MUT or no MUT, arm as MUT and no activity, arm as MUT, and after 1 min. human activity, arm as MUT, and 3 min after starting to eat honey. One can find absolutely two distinguish resonance frequencies with considerable change in different sampling conditions around $${f}_{01}=3.25$$ GHz and $${f}_{02}=4.67$$ GHz that be known as the first and the second resonance frequencies from Fig.6b, c, respectively. Figure 6b, c are depicted to have a precise analysis of the first and the second resonance frequency variation during the related arm MUT sampling change.

**Figure 7:**
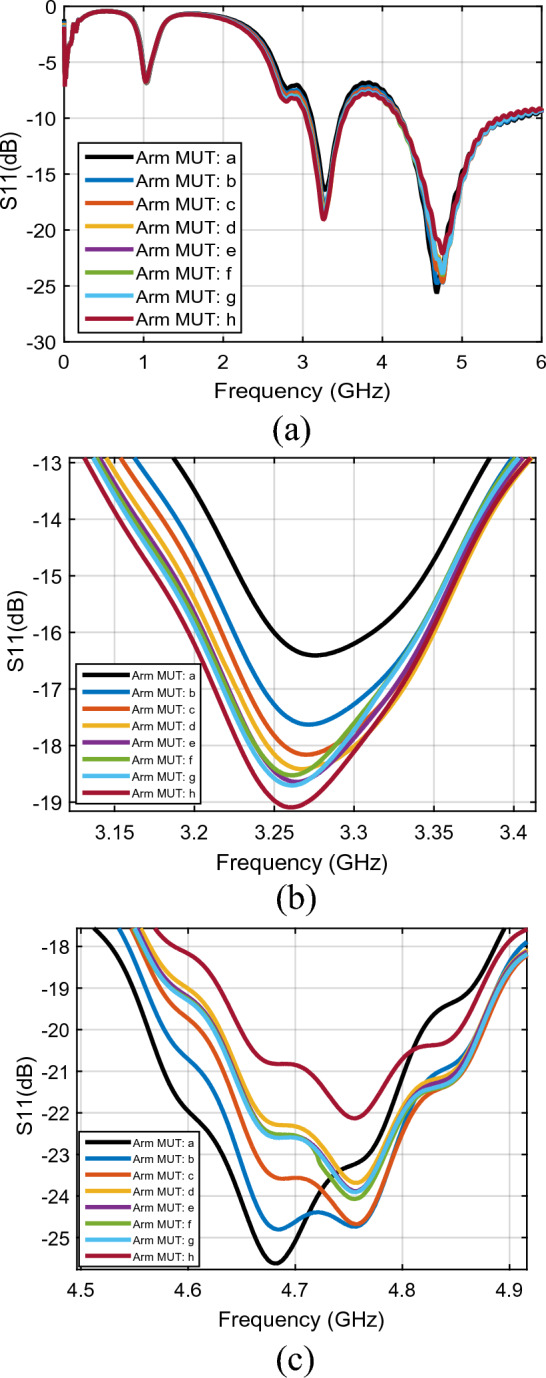
The return loss (${\mathrm{S}}_{11}$) per frequency for (**a**) first (${f}_{01}$) and second (${f}_{02}$) resonance frequencies. (**b**) First resonance frequency (${f}_{01}$). (**c**) Second resonance frequency (${f}_{02}$) (line a: arm as MUT with no activity, line **b**: after 1 min activity, line c: after 2 min activity, line d: after 3 min activity, line e: after 4 min activity, line f: after 4 min activity, line g: after 5 min activity, line h: after 6 min activity).

Figure 7a shows the measured performance of the PFR sensors for different MUTs over a wide frequency band (form DC to 6 GHz). The two resonance frequencies of the return loss of the sensor are reported to behave differently for the same MUT. As shown in Fig. 7b, c, the amplitude of ${S}_{11}$ is decreased at ${f}_{01}=3.25$ GHz (from − 16.4 to − 19.2 dB) while it is increased at ${f}_{02}=4.67$ GHz (from − 25.4 to −22.1 dB) under the same MUTs.

**Figure 8:**
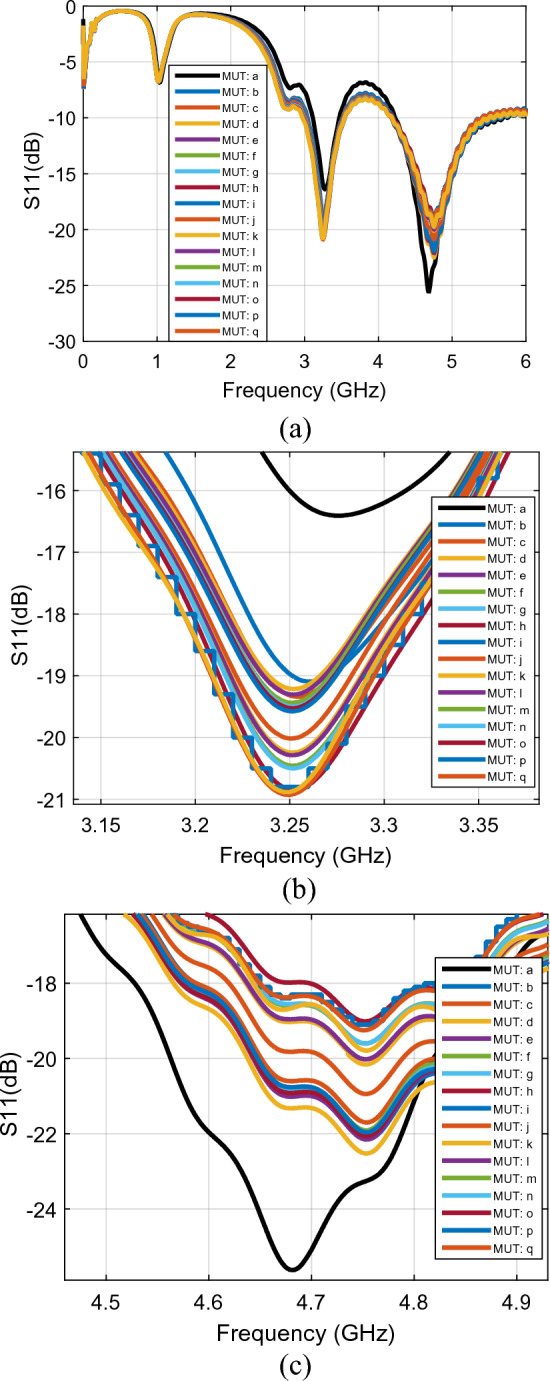
The return loss (${\mathrm{S}}_{11}$) per frequency for (**a**) first (${f}_{01}$) and second (${f}_{02}$) resonance frequencies. (**b**) First resonance frequency (${f}_{01}$). (**c**) Second resonance frequency (${f}_{02}$) (line a: arm as MUT with no activity, line b: after 6 min activity, line c: 3 min after starting eating honey, line d: 8 min after starting eating honey (eating ended), line e: 9 min, line f: 10 min , line g: 11 min , line h: 12 min , line i: 13 min , line j: 17 min , line k: , 18 min , line l: 21 min , line m: 23 min , line n: 33 min , line o: 36 min , line p: 43 min , line q: 57 min after eating honey.

In addition to activity, the PFR sensor is evaluated for blood glucose monitoring of the volunteers after eating a specific amount of honey due to the fact that honey changes the blood glucose rate. Figure 8a shows the measured performance of the PFR sensors for a specific amount of honey taken by the volunteers over the different amount of time as the different MUTs. The performance of the PFR sensor is measured over a wide frequency band (form DC to 6 GHz). Similar to the first case in Fig. 7, the two resonance frequencies of the return loss of the sensor are reported to behave differently for the same MUT. As shown in Fig. 8b, c, the amplitude of ${S}_{11}$ is decreased at ${f}_{01}=3.25$ GHz (from −16.3 to −20.96 dB) while it is increased at ${f}_{02}=4.67$ GHz (from −25.93 to − 18.73 dB) under the same MUTs.

The ${S}_{11}$ parameter of the PFR sensor is measured during the various conditions of samplings, i.e., in the resting, just after the volunteers’ warm-up, during the volunteers’ eating honey over the different time intervals as provided in the caption of Fig. 8. Investigation reveals that the amplitude of ${S}_{11}$ acording to different sampling condition is decraced due to human activity until a few minute after eating honey and increased again after that in the first resonance frequence; eating honey help to adjusting ${S}_{11}$.

**Figure 9:**
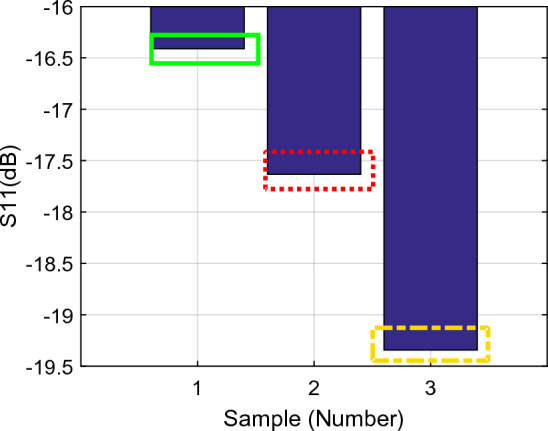
The return loss (${\mathrm{S}}_{11}$) per sample number at the first resonance frequency (${f}_{01}$), Sample1 (solid green rectangle), arm as MUT with no activity, Sample2 (dashed red rectangle), arm as MUT after activity, Sample3 (dashed yellow rectangle), and arm as MUT after eating honey*.*

**Figure 10:**
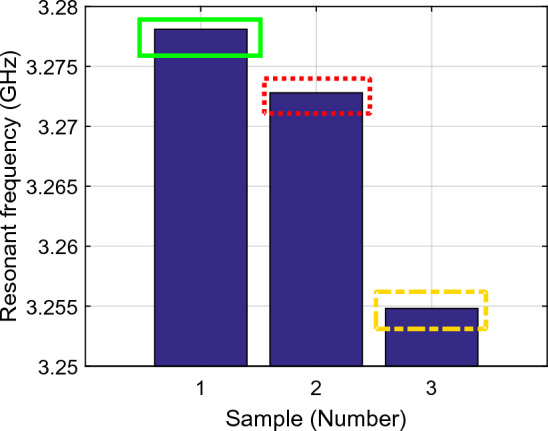
The resonance frequency per sample number at the first resonance frequency (${f}_{01}$), Sample1 (solid green rectangle), arm as MUT with no activity, Sample2 (dashed red rectangle), Arm as MUT after activity, Sample3 (dashed yellow rectangle), and arm as MUT after eating honey.

**Figure 11:**
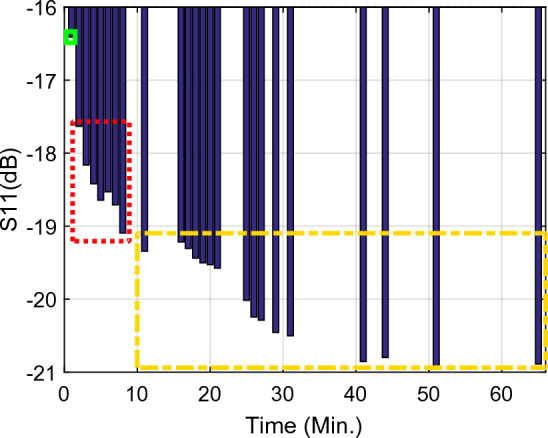
The return loss (${\mathrm{S}}_{11}$) per time at the first resonance frequency (${f}_{01}$) Sample1 (solid green rectangle): arm as MUT with no activity, Sample2 (dashed red rectangle), arm as MUT after activity, Sample3 (dashed yellow rectangle), and arm as MUT after eating honey.

**Figure 12:**
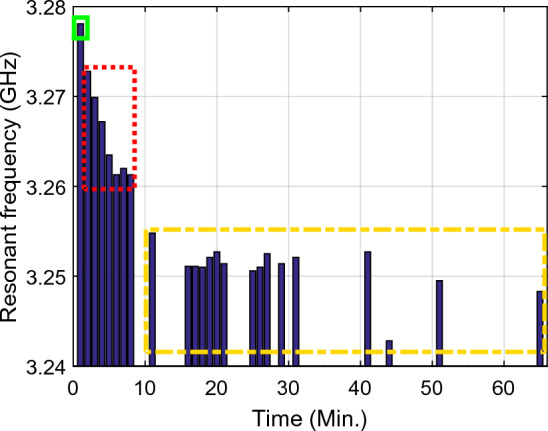
The resonance frequency per time at the first resonance frequency (${f}_{01}$), Sample1 (solid green rectangle), arm as MUT with no activity, Sample2 (dashed red rectangle), arm as MUT after activity, Sample3 (dashed yellow rectangle), and arm as MUT after eating honey.

**Figure 13:**
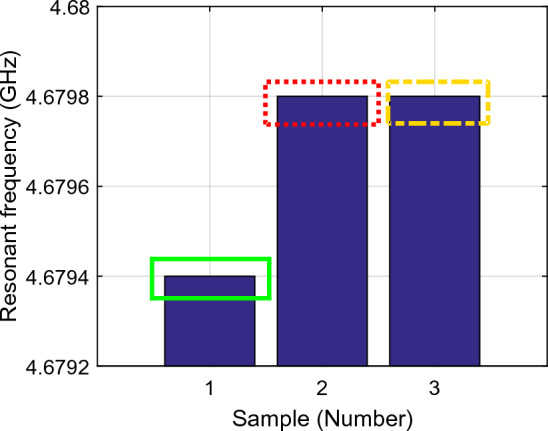
The resonance frequency per time at the second resonance frequency (${f}_{02}$) Sample1 (solid green rectangle), arm as MUT with no activity, Sample2 (dashed red rectangle), arm as MUT after activity, Sample3 (dashed yellow rectangle), and arm as MUT after eating honey.

**Figure 14:**
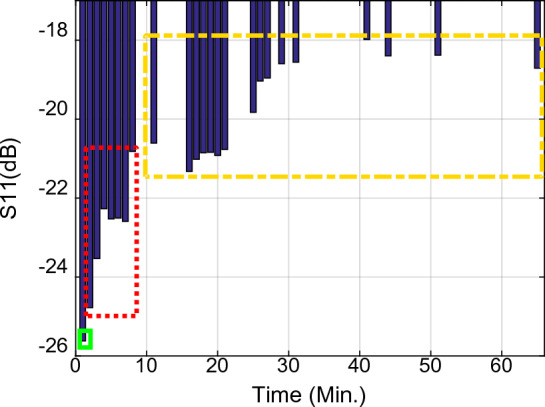
The return loss (${\mathrm{S}}_{11}$) per time at the second resonance frequency (${f}_{02}$) Sample1 (solid green rectangle), arm as MUT with no activity, Sample2 (dashed red rectangle), arm as MUT after activity, Sample3 (dashed yellow rectangle), and arm as MUT after eating honey.

**Figure 15:**
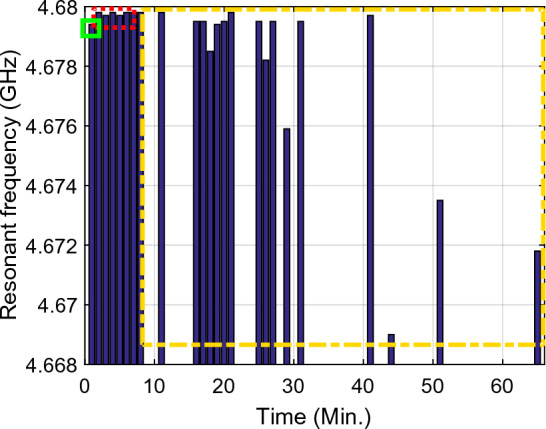
The resonance frequency per time at the second resonance frequency (${f}_{02}$), Sample1 (solid green rectangle), arm as MUT with no activity, Sample2 (dashed red rectangle), arm as MUT after activity, Sample3 (dashed yellow rectangle), and arm as MUT after eating honey.

Figure 9 shows the amplitude gain of the first resonance frequency (${f}_{01}$) during three parts of the sampling test, i.e., arm as MUT and no activity, arm as MUT after 6 min activity, and arm as MUT 3 min after starting to eat honey indicated as samples 1, 2 and 3 respectively. Similarly, Fig. 10 shows the related resonance frequencies correlated to the samples 1–3. The signal gains and resonance frequencies in different situations for the first resonance frequency are classified and exhibited in Figs. 11 and 12; i.e., arm as MUT with no activity, arm as MUT after human activity, arm as MUT and eating honey over the different time intervals. In addition, the same situations are considered for the second resonance frequency as provided in Figs. 13, 14 and 15.

Inspection of the measured results in Figs. 9, 10, 11, 12, 13, 14 and 15 reveals that similar to the nonlinear results in^60^ the trace of frequency change in the proposed sensor is not linear and depend on some factors, i.e., person with low- or high- fitness and measuring during or after exercise. Experimental results reveal that the blood glucose is changed due to the experiment condition changing. This recorded change by the designed sensor can be considered as an amplitude shifter in low frequency as well as a frequency shifter in higher frequency too.

It is worthy to mention that the on-body sweat of the patients and the slight movement of the PFR sensor can influence the accuracy of the measured results. Several factors potentially can influence the measured results of the sensor. Even in cases of volunteers exhibiting normal blood pressure, variables such as the thickness of the skin and fat layer, along with the volunteer's body temperature can influence the accuracy of the measured results. To mitigate the influence of external factors like environmental conditions, sweat, and contamination, a preliminary step is applied by cleansing the volunteer's skin with alcohol before placing the sensor on their arm. Moreover, it's noteworthy that the results might also be influenced by the volunteer's most recent meal which was consumed four hours prior to the testing.

## Conclusions and future works

In this paper, a non-invasive blood glucose monitoring EM-based sensor was designed and rapped on the human body. After that, a three-phase experiment was developed to monitor blood glucose change in the human body. The amplitude and frequency shifts are recorded due to blood glucose changes in lower frequency and higher frequency, respectively. Finally, the simulation results verify the efficiency of the proposed method. Future works can be directed to design a non-connected VNA sensor by developing a modulator circuit to this sensor to transmit the lower or higher frequency to be recorded in receiver devices like a smart watch, smart mobile phone, etc. Moreover, the PFR sensor can be used to predict the hypo- and hyperglycemia using machine learning techniques.

### Ethical approval

We confirm that all procedures were followed in accordance with the relevant guidelines and regulations. This study was reviewed and approved by the National Taiwan University Hospital Ethics Committee and issued a license document (case number: 202212043DINA) and the date 2023-02-14. We also confirm that all experimental protocols were authorized by the National Yunlin University of Science and Technology's Research Ethics Office (REO). The completion certificate has the number 202212213140 and the date 2022-12-21. In addition, we confirmed that informed consent was obtained from all subjects and/or their legal guardian(s) for experiments involving human participants.

## Data Availability

The datasets generated during and/or analysed during the current study are available from the corresponding author on reasonable request.

## References

[1] Salunkhe VA, Veluthakal R, Kahn SE, Thurmond DC (2018). Novel approaches to restore beta cell function in prediabetes and type 2 diabetes. Diabetologia.

[2] Chorbev I, Sotirovska M, Mihajlov D (2011). Virtual communities for diabetes chronic disease healthcare. Int. J. Telemed. Appl..

[3] Huang H-W (2022). An automated all-in-one system for carbohydrate tracking, glucose monitoring, and insulin delivery. J. Control. Release.

[4] Buford, R. J., Green, E. C. & McClung, M. J. A microwave frequency sensor for non-invasive blood-glucose measurement. In *2008 IEEE Sensors Applications Symposium*. 4–7 (IEEE, 2008).

[5] Choi, H., Luzio, S., Beutler, J. & Porch, A. Microwave noninvasive blood glucose monitoring sensor: Human clinical trial results. In *2017 IEEE MTT-S International Microwave Symposium (IMS)*. 876–879 (IEEE, 2017).

[6] Choi, H., Luzio, S., Beutler, J. & Porch, A. Microwave noninvasive blood glucose monitoring sensor: Penetration depth and sensitivity analysis. In *2018 IEEE International Microwave Biomedical Conference (IMBioC)*. 52–54 (IEEE, 2018).

[7] Caduff A, Dewarrat F, Talary M, Stalder G, Heinemann L, Feldman Y (2006). Non-invasive glucose monitoring in patients with diabetes: A novel system based on impedance spectroscopy. Biosens. Bioelectron..

[8] Tang F, Wang X, Wang D, Li J (2008). Non-invasive glucose measurement by use of metabolic heat conformation method. Sensors.

[9] Yadav J, Rani A, Singh V, Murari BM (2015). Comparative study of different measurement sites using NIR based non-invasive glucose measurement system. Proc. Comput. Sci..

[10] von Lilienfeld-Toal H, Weidenmüller M, Xhelaj A, Mäntele W (2005). A novel approach to non-invasive glucose measurement by mid-infrared spectroscopy: The combination of quantum cascade lasers (QCL) and photoacoustic detection. Vib. Spectrosc..

[11] Subramanian A, Adap S, Chawale S, Singh S, Sudhakaran P (2017). Non invasive glucose measurement using Raman spectroscopy. Int. Res. J. Eng. Technol. IRJET.

[12] Buchert, J.M. Thermal emission spectroscopy as a tool for noninvasive blood glucose measurements. In *Optical Security and Safety*. Vol. 5566. 100–111 (SPIE, 2004).

[13] Jang, S., Wang, Y. & Jang, A. Review of emerging approaches utilizing alternative physiological human body fluids in non-or minimally invasive glucose monitoring. In *Advanced Bioscience and Biosystems for Detection and Management of Diabetes*. 9–26 (Springer, 2022).

[14] Vashist SK, Zheng D, Al-Rubeaan K, Luong JH, Sheu F-S (2011). Technology behind commercial devices for blood glucose monitoring in diabetes management: A review. Anal. Chim. Acta.

[15] Sim JY, Ahn C-G, Jeong E-J, Kim BK (2018). In vivo microscopic photoacoustic spectroscopy for non-invasive glucose monitoring invulnerable to skin secretion products. Sci. Rep..

[16] Phan Q-H, Lai Y-R, Xiao W-Z, Lien C-H (2020). Surface plasmon resonance prism coupler for enhanced circular birefringence sensing and application to non-invasive glucose detection. Opt. Exp..

[17] Wang L (2020). Critical factors for in vivo measurements of human skin by terahertz attenuated total reflection spectroscopy. Sensors.

[18] Gabbay R, Sivarajah S, Wilkins R, Schurman M (2007). Optical coherence tomography-based continuous non-invasive glucose monitoring in patients with diabetes. Diabetes.

[19] Yeh S-J, Hanna CF, Khalil OS (2003). Monitoring blood glucose changes in cutaneous tissue by temperature-modulated localized reflectance measurements. Clin. Chem..

[20] Dantu, V., Vempati, J. & Srivilliputhur, S. Non-invasive blood glucose monitor based on spectroscopy using a smartphone. In *2014 36th Annual International Conference of the IEEE Engineering in Medicine and Biology Society*. 3695–3698 (IEEE, 2014). 10.1109/EMBC.2014.6944425 25570793

[21] Pickup JC, Hussain F, Evans ND, Rolinski OJ, Birch DJ (2005). Fluorescence-based glucose sensors. Biosens. Bioelectron..

[22] Habbu S, Dale M, Ghongade R (2019). Estimation of blood glucose by non-invasive method using photoplethysmography. Sādhanā.

[23] Domschke A, March WF, Kabilan S, Lowe C (2006). Initial clinical testing of a holographic non-invasive contact lens glucose sensor. Diabetes Technol. Ther..

[24] Kupcinskas RA (2000). A Method for Optical Measurement of Urea in Effluent Hemodialysate.

[25] Zafar H, Channa A, Jeoti V, Stojanović GM (2022). Comprehensive review on wearable sweat-glucose sensors for continuous glucose monitoring. Sensors.

[26] Yunos MFAM, Nordin AN (2020). Non-invasive glucose monitoring devices: A review. Bull. Electr. Eng. Inform..

[27] Sempionatto JR (2019). Eyeglasses-based tear biosensing system: Non-invasive detection of alcohol, vitamins and glucose. Biosens. Bioelectron..

[28] Freer B (2011). Feasibility of a Non-Invasive Wireless Blood Glucose Monitor.

[29] Leboulanger B, Guy RH, Delgado-Charro MB (2004). Reverse iontophoresis for non-invasive transdermal monitoring. Physiol. Meas..

[30] Meyhöfer S (2020). Evaluation of a near-infrared light ultrasound system as a non-invasive blood glucose monitoring device. Diabetes Obes. Metab..

[31] Choi H (2015). Design and in vitro interference test of microwave noninvasive blood glucose monitoring sensor. IEEE Trans. Microwave Theory Tech..

[32] Liu, L.W. *et al.* Portable and non-invasive blood glucose monitoring over a prolonged period using whispering gallery modes at 2.4 GHz. In *2020 IEEE Ukrainian Microwave Week (UkrMW)*. 599–602 (IEEE, 2020).

[33] Liu LW, Kandwal A, Cheng Q, Shi H, Tobore I, Nie Z (2019). Non-invasive blood glucose monitoring using a curved Goubau line. Electronics.

[34] Liu LW (2020). In-vivo and ex-vivo measurements of blood glucose using whispering gallery modes. Sensors.

[35] Kim J, Rahmat-Samii Y (2004). Implanted antennas inside a human body: Simulations, designs, and characterizations. IEEE Trans. Microwave Theory Tech..

[36] C. M. Studio. *C st Studio Suite 2013.* (Computer Simulation Technology AG, 2013).

[37] Durney, C., Massoudi, H. & Iskander, M. *Radiofrequency Radiation Dosimetry Handbook*. (Brooks Air Force Base, US Air Force School of Aerospace, Medical Division; Reg. No. SAM-TR-85-731985).

[38] Ahlbom A (1998). Guidelines for limiting exposure to time-varying electric, magnetic, and electromagnetic fields (up to 300 GHz). Health Phys..

[39] Hayati M (2009). Loaded coupled transmission line approach of left-handed (LH) structures and realization of a highly compact dual-band branch-line coupler. Prog. Electromag. Res. C.

[40] Nosrati, M. *et al*. A novel ultra wideband (UWB) filter with double tunable notch-bands using MEMS capacitors. In: *IEEE MTT-S Microwave Symposium*. 1–3 (2013).

[41] Nosrati A, Mohammad-Taheri M, Nosrati M (2020). Back-to-back aperture-and gap-coupled discontinuities integration for band-pass filter design. Electr. Lett..

[42] Nosrati A, Mohammad-Taheri M, Nosrati M (2020). Gap-coupled dual-band evanescent-mode substrate integrated band-pass filter waveguide. Prog. Electromagnet. Res. Lett..

[43] Nosrati, M., Soltanian, F. & Nosrati, A. Size reduction and performance enhancement of coplanar-waveguide resonators for surface plasmonic applications. In: *IEEE Transactions on Components, Packaging and Manufacturing Technology*. Vol. 12(12). 1995–2001 (2022).

[44] Kiani, S., Rezaei, P. & Fakhr, M. Dual-frequency microwave resonant sensor to detect noninvasive glucose-level changes through the fingertip. In *IEEE Transactions on Instrumentation and Measurement*. Vol. 70(art no. 6004608). 1–8 (2021). 10.1109/TIM.2021.3052011.

[45] Kiani S, Rezaei P, Karami M, Sadeghzadeh RA (2019). Band-stop filter sensor based on SIW cavity for the non-invasive measuring of blood glucose. IET Wirel. Sens. Syst..

[46] Kim Y, Salim A, Lim S (2021). Millimeter-wave-based spoof localized surface plasmonic resonator for sensing glucose concentration. Biosensors.

[47] Dai LH, Zhao HZ, Zhao X, Zhou YJ (2020). Flexible and printed microwave plasmonic sensor for noninvasive measurement. IEEE Access.

[48] Wang Z (2022). Tunable refractive index sensor made using graphene with a high figure of merit. J. Appl. Spectrosc..

[49] Zafar R, Salim M (2015). Enhanced figure of merit in Fano resonance-based plasmonic refractive index sensor. IEEE Sens. J..

[50] Rakhshani MR (2019). Fano resonances based on plasmonic square resonator with high figure of merits and its application in glucose concentrations sensing. Opt. Quantum Electron..

[51] Zangeneh-Nejad F, Fleury R (2019). Topological Fano resonances. Phys. Rev. Lett..

[52] Muñoz-Enano J, Vélez P, Su L, Gil M, Casacuberta P, Martín F (2020). On the sensitivity of reflective-mode phase-variation sensors based on open-ended stepped-impedance transmission lines: Theoretical analysis and experimental validation. IEEE Trans. Microwave Theory Tech..

[53] Kiani S, Rezaei P, Navaei M (2020). Dual-sensing and dual-frequency microwave SRR sensor for liquid samples permittivity detection. Measurement.

[54] Kiani S, Rezaei P, Navaei M, Abrishamian MS (2018). Microwave sensor for detection of solid material permittivity in single/multilayer samples with high quality factor. IEEE Sens. J..

[55] Navaei M, Rezaei P, Kiani S (2023). A symmetric bar chart-shape microwave sensor with high Q-factor for permittivity determination of fluidics. Int. J. Microwave Wirel. Technol..

[56] Shen X, Cui TJ (2014). Ultrathin plasmonic metamaterial for spoof localized surface plasmons. Laser Photon. Rev..

[57] Zhang X, Yan RT, Cui TJ (2020). High-FoM resonance in single hybrid plasmonic resonator via electromagnetic modal interference. IEEE Trans. Antennas Propag..

[58] Delavaud, C., Ciais, P., Wai Po, F.C., de Foucauld, E. & David, J.-B. *Modern Telemetry*. 223–246 (2011).

[59] Wriggers P, Lenarz T (2017). Biomedical Technology: Modeling, Experiments and Simulation.

[60] Tyler NS, Lopez CM, Young GM, El Youssef J, Castle JR, Jacobs PG (2022). Quantifying the impact of physical activity on future glucose trends using machine learning. iScience.

